# β,γ-Methylene-ATP and its metabolite medronic acid affect both arterial media calcification and bone mineralization in non-CKD and CKD rats

**DOI:** 10.1093/jbmrpl/ziae057

**Published:** 2024-04-22

**Authors:** Britt Opdebeeck, Astrid Van den Branden, Saar Adriaensen, Isabel R Orriss, Jessal J Patel, Hilde Geryl, Kathleen Zwijsen, Patrick C D’Haese, Anja Verhulst

**Affiliations:** Laboratory of Pathophysiology, Department of Biomedical Sciences, University of Antwerp, Antwerp 2610, Belgium; Laboratory of Pathophysiology, Department of Biomedical Sciences, University of Antwerp, Antwerp 2610, Belgium; Laboratory of Pathophysiology, Department of Biomedical Sciences, University of Antwerp, Antwerp 2610, Belgium; Department of Comparative Biomedical Science, Royal Veterinary College, London NW1 0TU, United Kingdom; Department of Comparative Biomedical Science, Royal Veterinary College, London NW1 0TU, United Kingdom; Laboratory of Pathophysiology, Department of Biomedical Sciences, University of Antwerp, Antwerp 2610, Belgium; Laboratory of Experimental Medicine and Pediatrics, Inflamed Center of Excellence, University of Antwerp, Antwerp 2610, Belgium; Laboratory of Pathophysiology, Department of Biomedical Sciences, University of Antwerp, Antwerp 2610, Belgium; Laboratory of Pathophysiology, Department of Biomedical Sciences, University of Antwerp, Antwerp 2610, Belgium

**Keywords:** arterial calcification, bone matrix mineralization, extracellular nucleotides, ectonucleotidases, chronic kidney disease, warfarin, animal model

## Abstract

Arterial media calcification or pathological deposition of calcium-phosphate crystals in the vessel wall contributes significantly to the high mortality rate observed in patients with CKD. Extracellular nucleotides (ie, ATP or UTP) regulate the arterial calcification process by interacting with (1) purinergic receptors and (2) breakdown via ecto-nucleotidases, such as ectonucleotide pyrophosphatase/phosphodiesterase NPP1 or NPP3, affecting the local levels of calcification inhibitor, pyrophosphate, and stimulator inorganic phosphate (PP_i_/P_i_ ratio). Also, it has been shown that ATP analogs (ie, β,γ-methylene-ATP [β,γ-meATP]) inhibit vascular smooth muscle cell calcification in vitro. In the first experiment, daily dosing of β,γ-meATP (2 mg/kg) was investigated in rats fed a warfarin diet to trigger the development of non–CKD-related arterial medial calcifications. This study showed that β,γ-meATP significantly lowered the calcium scores in the aorta and peripheral vessels in warfarin-exposed rats. In a second experiment, daily dosing of 4 mg/kg β,γ-meATP and its metabolite medronic acid (MDP) was analyzed in rats fed an adenine diet to promote the development of CKD-related arterial medial calcification. Administration of β,γ-meATP and MDP did not significantly decrease aortic calcification scores in this model. Moreover, both compounds induced deleterious effects on physiological bone mineralization, causing an imminent risk for worsening the already compromised bone status in CKD. Due to this, it was not possible to raise the dosage of both compounds to tackle CKD-related arterial calcification. Again, this points out the difficult task of targeting solely ectopic calcifications without negatively affecting physiological bone mineralization. On the other hand, aortic mRNA expression of *Enpp1* and *Enpp3* was significantly and positively associated with aortic calcification scores, suggesting that normalizing the aortic NPP1/3 activity to control values might be a possible target to treat (CKD-induced) arterial media calcifications.

## Introduction

Arterial media calcification, also called Mönckeberg’s arteriosclerosis, can be defined as an ectopic deposition of calcium-phosphate (hydroxyapatite) crystals in the medial layer of medium- to large-sized arteries. Arterial media calcification contributes significantly to cardiovascular mortality in the elderly and patients with CKD, diabetes, and osteoporosis.^1-3^ Statements about arterial media calcification such as “The silent killer of the leg” or “The killer of patients with CKD” have been made.^3^^,^^4^ Patients with arterial media calcification have poor clinical outcomes, since they develop hypertension, arterial stiffness, and left ventricular hypertrophy, ultimately leading to heart failure and impaired coronary perfusion.^5^ The dual etiology of arterial calcification is (1) a passive precipitation of saturated calcium and phosphate in the vessel wall and (2) an active, cell-regulated process.^6^ Vascular smooth muscle cells (VSMCs) reside in the medial layer of the vessel wall and maintain arterial compliance (vasodilation and constriction). During the arterial media calcification process, an imbalance of calcification inhibitors (ie, pyrophosphate [PP_i_], matrix Gla protein [MGP], and fetuin-A) and stimulators (ie, hyperphosphatemia, hypercalcemia, and uremic toxins) is observed. This stimulates the VSMCs to undergo a phenotypic switch into more osteoblast-like cells, which leads to upregulation of osteo/chondrogenic marker genes such as *Runx2*, tissue nonspecific alkaline phosphatase (*Alpl*), *BMP2*, and SRY-Box Transcription Factor 9 (*Sox9*). These calcifying VSMCs produce and release mineralization-competent matrix vesicles, in which calcium and inorganic phosphate (P_i_) are built up, into the extracellular matrix, resulting in mineralization of the arterial wall.^6^^,^^7^ Subsequently, the pathological process of arterial media calcification resembles the process of physiological bone mineralization.^8^

Previous studies by Orriss et al^9-13^ have shown that extracellular nucleotides such as ATP and UTP influence the bone mineralization process either (1) via their hydrolysis product PP_i_, which binds to hydroxyapatite crystals and prevents further incorporation of phosphate into the crystals, or (2) by activation of purinergic P2 receptors. Given the similarities between arterial calcification and physiological bone mineralization, interest in the potential role of extracellular nucleotides in preventing arterial calcifications has emerged.

Extracellular nucleotides bind to purinergic receptors consisting of 2 broad family types: P1 and P2 receptors. The G protein–coupled P1 receptor family contains 4 subtypes (A_1_, A_2A_, A_2B_, and A_3_) and are activated by adenosine. The P2 receptor family can be further subdivided into 8 G protein–coupled P2Y receptors (P2Y_1,2,4,6,11,12,13,14_) and 7 ligand-gated ion channel P2X receptors (P2X1–7).^14^ Interestingly, the mRNA expression of the P2X1, P2X2, P2X4, P2X5, P2X6, and P2Y_2_ receptors was increased up to 3-fold in calcifying VSMCs compared with control VSMCs, suggesting a potential role in ectopic mineralization.^15^ Moreover, Patel et al^16^ showed that P2X receptor agonists βz-ATP, α,β-methylene-ATP (α,β-meATP), and β,γ-methylene-ATP (β,γ-meATP) inhibit VSMC calcification in vitro by diminishing VSMC apoptosis and VSMC phenotypic changes.

This study evaluated, in the first instance, whether the ATP analog and P2X1/P2X3 receptor agonist β,γ-meATP is able to prevent the development of arterial media calcification in a warfarin rat model. Warfarin belongs to the anticoagulant therapies and interferes with vitamin K epoxide reductase, antagonizing vitamin K recycling.^17^ However, the activation of the calcification inhibitor MGP depends on vitamin K. Hence, high concentrations of warfarin favor the development of arterial media calcifications. Rats exposed to a warfarin-containing diet experience a gradual time-dependent increase in calcium content in the aorta and peripheral arteries after 6 weeks and onwards.^18^ This rat model of warfarin-induced arterial media calcification allows us to investigate the potential direct therapeutic effect of β,γ-meATP on the process of arterial media calcification without the interference of CKD-, diabetes-, or osteoporosis-related risk factors. Additionally, this study investigated whether β,γ-meATP and its metabolite medronic acid (MDP) halted the development of CKD-related arterial media calcification by the use of an adenine-induced CKD rat model.

## Materials and methods

### Animal experiments

All animal experiments were performed in accordance with the National Institutes of Health Guide for the Care and Use of Laboratory Animals 85-23 (1996) and approved by the University of Antwerp Ethics Committee (permit number: 2017-05). Animals were housed 2 per cage, exposed to 12-hour light/dark cycles, and had free access to food and water.

#### Warfarin (non-CKD)–induced arterial media calcification rat study

To induce arterial calcification, 20 male Wistar rats (200–250 g; Iffa Credo, Belgium) were administered a warfarin-containing diet (0.30% warfarin and 0.15% vitamin K_1_ to prevent lethal bleeding; synthetic diet from SSNIFF Spezialdiäten, Soest, Germany) for the entire study period and were subjected to the following treatments: (1) vehicle (*n* = 10) or (2) 2 mg/kg/day β,γ-meATP (*n* = 10). Vehicle and β,γ-meATP were administered daily via i.p. injections. Eight male Wistar rats were included as control animals that were fed a standard feed-pellet diet and daily i.p. injections of vehicle (PBS). Two animals (control rat and β,γ-meATP–treated rat) died before the planned sacrifice at week 8 and were not taken into account for further analyses.

#### CKD-induced arterial media calcification rat study

To induce CKD, a high-phosphate (1.2%) diet (synthetic diet from SSNIFF Spezialdiäten, Soest, Germany) for 2 weeks followed by a 0.75% adenine diet (synthetic diet from SSNIFF Spezialdiäten) for 4–5 weeks was administered to 36 male Wistar rats. The rats were randomly assigned to 3 study groups: (1) CKD rats treated with vehicle (*n* = 12), (2) CKD rats treated with 4 mg/kg/day β,γ-meATP, and (3) CKD rats treated with 4 mg/kg/day MDP. All treatments were given via i.p. injections and started together with adenine exposure. Ten male Wistar rats were included as control animals who received a standard feed-pellet diet and daily i.p. injections of vehicle (PBS solution).

Before the start of the studies, at week 4, and at sacrifice, blood sampling was performed via the tail vein of restraint animals. At sacrifice, rats were exsanguinated through the retro-orbital plexus after anesthesia with 80 mg/kg ketamine (Pfizer, Puurs, Belgium) and 10 mg/kg xylazine (Bayer Animal Health, Monheim, Germany) via i.p. injection.

### Biochemical analysis

Total serum phosphorus levels were analyzed with the Ecoline S Phosphate kit (Diasys, Holzheim, Germany) and serum calcium levels were determined with flame atomic absorption spectrometry (Perkin-Elmer, Wellesley, MA, USA) after appropriate dilution in 0.1% La(NO_3_)_3_ to eliminate chemical interference during analysis. Serum creatinine levels were evaluated according to the Jaffé method.^19^

### Quantification of arterial media calcification

To visualize and quantify calcification in the aortic wall, sections of the thoracic aorta were stained using Von Kossa’s method. Following isolation, the thoracic aorta was fixed in neutral buffered formalin for 90 minutes. It was then cut into 15–20 segments of 2 to 3 mm which were embedded upright in a paraffin block. Four-micrometer-thick sections were stained with Von Kossa’s and counterstained with hematoxylin and eosin to detect and quantify calcification. The percentage of calcified area was calculated using Axiovision image analysis software (release 4.5; Carl Zeiss, Oberkochen, Germany), in which 2 color-separation thresholds measure the total tissue area and the Von Kossa positive area. After summing both absolute areas, the percentage of calcified area was calculated as the ratio of the Von Kossa positive area versus the total tissue area. Calcification in the aorta and smaller arteries was also quantified by measurement of the calcium content. The proximal part of the abdominal aorta and the left carotid and femoral arteries were isolated and directly weighed on a precision balance. The arterial samples were digested in 65% HNO_3_ at 60°C for 6 hours. The calcium content in the digested samples was measured with flame atomic absorption spectrometry and expressed as milligrams of calcium/gram of wet tissue.

### Quantitative real-time PCR

The mRNA transcript expression of glyceraldehyde 3-phosphate dehydrogenase (*Gapdh, OMIM 138400*) (Rn99999916_s1), *Sox9, OMIM 608160* (Rn01751069_mH), *Enpp1, OMIM 173335* (Rn01638706_m1), *Enpp3, OMIM 602182* (Rn00571329_m1), *Alpl*, *OMIM 171760* (Rn01516028_m1), and *P2rx1, OMIM 600845* (Rn00564454_m1) was determined in the distal part of the abdominal aorta. Total mRNA was extracted using the Isolate II RNA mini kit (Bioline Meridian Bioscience, Memphis, TN, USA) and reverse-transcribed to cDNA by the High Capacity cDNA archive kit (Applied Biosystems, Foster City, CA, USA). Real-time PCR with a QuantStudio 3 Detection System (ThermoFisher Scientific) based on TaqMan fluorescence methodology was used for mRNA quantification. TaqMan probe and primers were purchased as TaqMan gene expression assays-on-demand from ThermoFisher Scientific. Each gene was tested in triplicate and normalized to the expression of the housekeeping transcript GAPDH.

### Bone histomorphometry

The left tibia was fixed in 70% ethanol overnight at 4°C, dehydrated, and embedded in 100% methylmethacrylate (Merck, Hohenbrunn, Germany) for bone histomorphometry. For analysis of static bone parameters, 5-μm-thick sections were Goldner stained for visualization and measurement of total bone area, mineralized bone area, osteoid area, eroded perimeter, and osteoblast and osteoclast perimeter using the Axiovision image analysis software (release 4.5; Carl Zeiss).

### Statistical analysis

Statistical comparisons were made by nonparametric testing (Prism 8.1.1; GraphPad Software, Inc, La Jolla, CA, USA). To investigate the statistical difference between groups at 1 time point, a Kruskal-Wallis test multiple comparisons was applied. The *p*-value was adjusted by Bonferroni correction. To investigate the relationship between aortic calcification and aortic NPP1 or NPP3 mRNA expression, a Spearman’s rho univariate correlation analysis was performed. Representative data in tables are presented as median ± IQR. Representative data in graphs are presented as individual values and medians. Data are considered significant when the adjusted *p*-value ≤ .05.

## Results

### β,γ-meATP and MDP had no major effects on serum calcium, phosphorus, and creatinine levels in both the warfarin- and adenine-induced arterial media calcification rat models

#### Warfarin (non-CKD)–induced arterial media calcification rat study

As serum calcium and phosphorus are strong inducers of the arterial calcification process, the effect of daily β,γ-meATP administration on circulating calcium and phosphorus levels was investigated in rats exposed to warfarin. No difference between groups was observed for serum phosphorus levels; however, a small but significant decrease in serum calcium levels was found for β,γ-meATP–treated rats exposed to warfarin versus control rats fed a standard feed-pellet diet. Serum creatinine, a marker of renal function, remained unchanged between all groups (Table 1).

**Table 1:** Serum biochemical parameters of warfarin-exposed rats at sacrifice.

	**Control (normal diet)**	**Vehicle (warfarin diet)**	**β,γ-meATP (warfarin diet)**
Calcium (mg/dL)	14.9 ± 0.8	13.4 ± 2.0	12.3 ± 1.3^*^
Phosphorus (mg/dL)	9.0 ± 3.8	9.3 ± 3.4	10.0 ± 3.2
Creatinine (mg/dL)	0.8 ± 0.3	0.9 ± 0.3	0.9 ± 0.2

Data are presented as median ± IQR. ^*^p < .05 vs control. Control (*n* = 8), vehicle (*n* = 10), and β,γ-meATP (*n* = 9). Abbreviation: β,γ-meATP, β,γ-methylene-ATP.

#### CKD-induced arterial media calcification rat study

Chronic renal failure was effectively induced via administration of a 0.75% adenine diet, as evidenced by a significant increase in serum phosphorus and creatinine levels in all CKD groups versus rats fed a standard feed-pellet diet (control). Both treatments, β,γ-meATP and MDP, did not alter serum calcium, phosphorus, and creatinine levels (Table 2).

**Table 2:** Serum biochemical parameters of CKD-exposed rats at sacrifice.

	**Control (normal diet)**	**Vehicle (CKD diet)**	**β,γ-meATP (CKD diet)**	**MDP (CKD diet)**
Calcium (mg/dL)	12.3 ± 2.6	13.1 ± 0.7	12.6 ± 1.1	13.0 ± 0.9
Phosphorus (mg/dL)	8.1 ± 2.3	13.2 ± 6.1^*^^*^	13.6 ± 3.8^*^^*^	14.1 ± 4.8^*^^*^^*^
Creatinine (mg/dL)	0.9 ± 0.3	2.3 ± 1.5^*^^*^	2.7 ± 1.6^*^^*^^*^	3.3 ± 1.4^*^^*^^*^^*^

Data are presented as median ± IQR. ^*^^*^p < .01, ^*^^*^^*^p < .001, and ^*^^*^^*^^*^p < .0001 vs control. Control (*n* = 10), vehicle (*n* = 12), β,γ-meATP (*n* = 11), and MDP (*n* = 12). Abbreviations: MDP, medronic acid; β,γ-meATP, β,γ-methylene-ATP.

### Administration of β,γ-meATP significantly reduced calcification in the aorta and peripheral vessels of warfarin-exposed rats, but not in CKD-exposed rats

#### Warfarin (non-CKD)–induced arterial media calcification rat study

**Figure 1:**
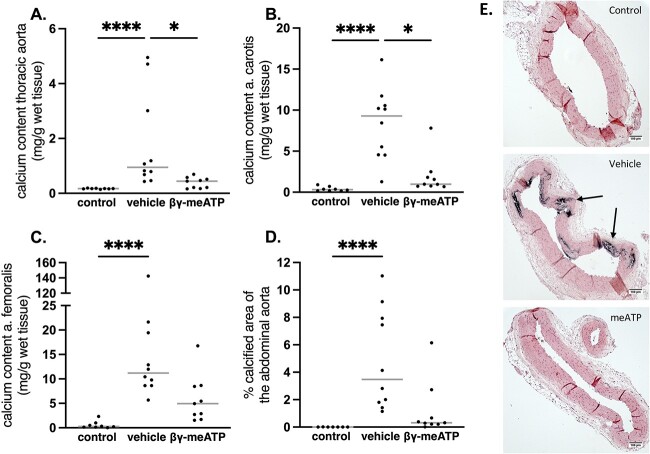
The effect of daily treatment with β,γ-methylene-ATP (β,γ-meATP) on warfarin-induced calcification in the aorta and peripheral arteries. Calcium contents of the (A) thoracic aorta, (B) carotid artery, and (C) femoral artery and (D) the percentage of calcified aortic area. Data are presented as individual values (dots) and median (gray lines). ^*^p < .05, ^*^^*^^*^^*^p < .001. Significant effect between vehicle and β,γ-meATP for calcium content in the femoral artery (*p* = .04) and % calcified area of the abdominal aorta (*p* = .035) is reached when no Bonferroni correction was applied. (E) Representative Von Kossa–stained aortic sections. Arrows point toward calcification sites. Vehicle (*n* = 10) and β,γ-meATP (*n* = 9).

The total calcium content was analyzed in the thoracic aorta and the carotid and femoral artery as a measure for the presence of arterial media calcification. Exposure to warfarin induced a significant increase in the calcium content in the different vessels. The β,γ-meATP–treated rats had a significantly lower calcium content in the thoracic aorta and carotid arteries compared with vehicle-treated rats (Figure 1A and B). Although not significant, a decreasing trend is shown for the calcium content of the femoral arteries in β,γ-meATP–treated rats versus vehicle-treated rats (Figure 1C). Likewise, a reduction in the calcified area in the abdominal aorta of β,γ-meATP–treated rats, as indicated by Von Kossa staining, was observed (Figure 1D). Von Kossa staining also revealed that calcifications were solely present in the arterial media layer (Figure 1E).

#### CKD-induced arterial media calcification rat study

**Figure 2:**
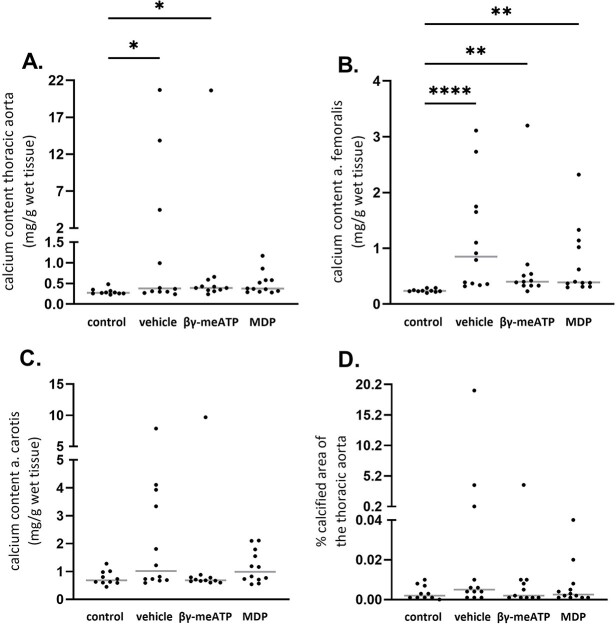
The effect of daily treatment with β,γ-methylene-ATP (β,γ-meATP) or medronic acid (MDP) on CKD-related calcification in the aorta and peripheral arteries. Calcium contents of the (A) thoracic aorta, (B) femoral artery, and (C) carotid artery and (D) the percentage of calcified aortic area. Data are presented as individual values (dots) and median (gray lines). ^*^p < .05, ^*^^*^p < .01, and ^*^^*^^*^^*^p < .0001. Control (*n* = 10), vehicle (*n* = 12), β,γ-meATP (*n* = 11), and MDP (*n* = 12).

Induction of CKD significantly increased the calcium content in the thoracic aorta and femoral and carotid arteries as well as the percentage of calcified aortic area versus control rats (Figure 2). Based on aortic calcification scores, β,γ-meATP and its metabolite MDP showed a trend towards lower calcium scores, although this did not reach statistical significant.

### β,γ-meATP and MDP abolish the upregulation of *Sox9* aortic mRNA expression (an osteo-/chondrogenic marker), mediated by the presence of arterial media calcification, in adenine-exposed rats

#### Warfarin (non-CKD)–induced arterial media calcification rat study

**Figure 3:**
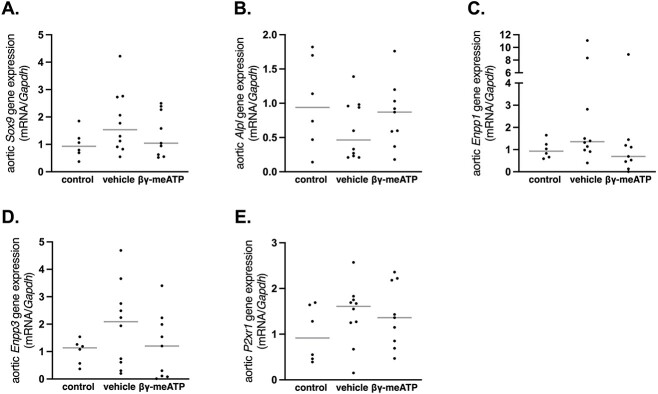
The effect of β,γ-methylene-ATP (β,γ-meATP) on the aortic mRNA expression of genes involved in the osteo-/chondrogenic trans-differentation, the PPi/Pi ratio, and the P2X1 receptor gene in warfarin-exposed rats. Total mRNA expression of (A) *Sox9*, (B) *Alpl*, (C) *Enpp1*, (D) *Enpp3*, and (E) *P2xr1*. Data are presented as individual values (dots) and median (gray lines). Control (*n* = 6), vehicle (*n* = 10), and β,γ-meATP (*n* = 9). Abbreviations: PPi/Pi ratio, pyrophosphate/inorganic phosphate ratio

The aortic mRNA expression of genes involved in the trans-differentation towards osteo-/chondrogenic cells, including *Sox9* and *Alpl* (encoding TNAP) and the pyrophosphate/inorganic phosphate (PP_i_/P_i_ ) ratio including *Enpp1* and *Enpp3,* were analyzed. Since β,γ-meATP is a P2X1 receptor agonist, its mRNA expression in the aorta was also investigated. As shown in Figure 3, no significant difference between groups was found for the aortic mRNA expression of the *Sox9, Alpl* (encoding TNAP), *Enpp1*, *Enpp3*, and *P2rx1* (encoding P2X1 receptor). However, a trend was observed wherein treatment with β,γ-meATP returned aortic mRNA expression levels of the different genes to those seen in control rats.

#### CKD-induced arterial media calcification rat study

**Figure 4:**
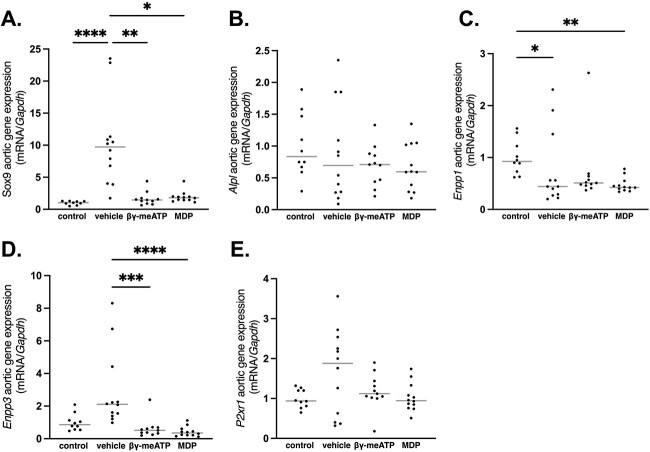
The effect of β,γ-methylene-ATP (β,γ-meATP) and medronic acid (MDP) on the aortic mRNA expression of genes involved in the osteo-/chondrogenic trans-differentation, the PPi/Pi ratio, and the P2X1 receptor gene in chronic renal failure rats. Total mRNA expression of (A) *Sox9*, (B) *Alpl*, (C) *Enpp1*, (D) *Enpp3*, and (D) *P2xr1*. Data are presented as individual values (dots) and median (gray lines). ^*^p < .05, ^*^^*^p < .01, ^*^^*^^*^p < .001, and ^*^^*^^*^^*^p < .0001. Control (*n* = 10), vehicle (*n* = 12), β,γ-meATP (*n* = 11), and MDP (*n* = 12).

**Figure 5:**
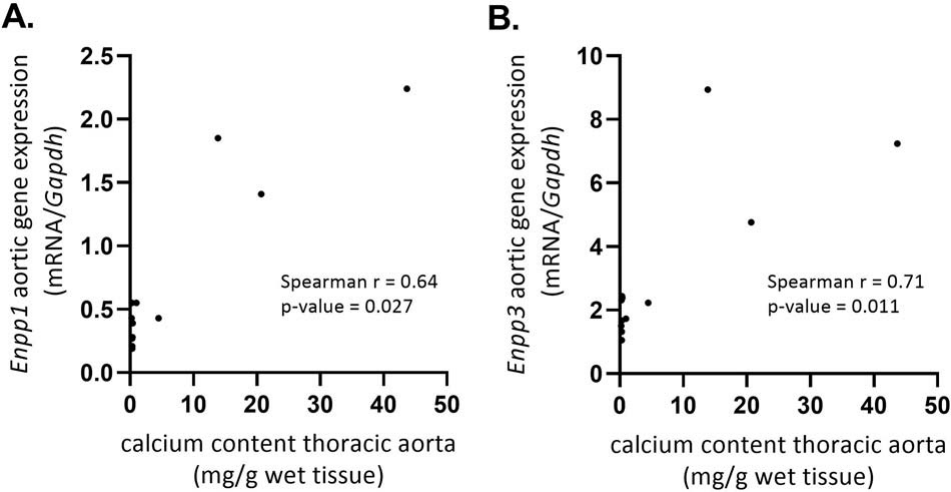
The correlation between calcium content scores in the thoracic aorta and *Enpp1* and *Enpp3* aortic mRNA gene expression in vehicle-treated CKD rats. A positive correlation of the calcium content in the thoracic aorta (mg/g wet tissue) versus (A) *Enpp1* and (B) *Enpp3* mRNA gene expression in the aorta of vehicle-treated CKD rats. Vehicle (*n* = 12).

Figure 4 shows that treating CKD rats either with β,γ-meATP or MDP significantly inhibited the mRNA upregulation of the osteo-/chondrogenic marker gene *Sox9,* as compared with vehicle-treated CKD rats, while no differences between groups were observed for the early osteo-/chondrogenic marker gene *Alpl* (encoding TNAP). Furthermore, vehicle-treated CKD rats had significantly lower aortic *Enpp1* mRNA expression as compared with control rats. This effect became even stronger after treatment with MDP. Conversely, *Ennp3* aortic mRNA expression increased in vehicle-treated CKD rats, while treatment with either β,γ-meATP or MDP abolished this effect. Furthermore, a positive and significant correlation between aortic calcium scores and *Enpp1* (Spearman’s *r* = 0.64, *p* = .027) or *Enpp3* (Spearman’s *r* = 0.71, *p* = .011) aortic mRNA expression was found for vehicle-treated CKD rats (Figure 5). With regard to *P2xr1* aortic mRNA gene expression, no differences between the groups were observed (Figure 4).

### β,γ-meATP and MDP affect physiological bone mineralization in both the warfarin- and adenine-induced arterial media calcification rat models.

#### Warfarin (non-CKD)–induced arterial media calcification rat study

**Figure 6:**
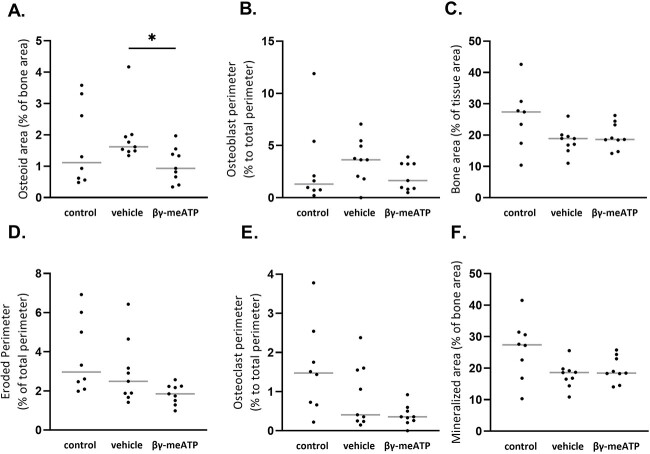
The effect of daily treatment with β,γ-methylene-ATP (β,γ-meATP) treatment on static bone parameters in warfarin-exposed rats. (A) Osteoid area, (B) osteoblast perimeter, (C) bone area, (D) eroded perimeter, (E) osteoclast perimeter, and (F) mineralized area. Data are presented as individual values (dots) and median (gray lines). ^*^p < .05. Control (*n* = 8), vehicle (*n* = 9), and β,γ-meATP (*n* = 9).

Exposure to warfarin does not significantly influence static bone parameters osteoid and eroded area, osteoblast and osteoclast perimeter, and bone and mineralized area versus control rats. However, daily treatment with β,γ-meATP significantly lowered the percentage of osteoid area compared with that in vehicle-treated rats (Figure 6).

#### CKD-induced arterial media calcification rat study

**Figure 7:**
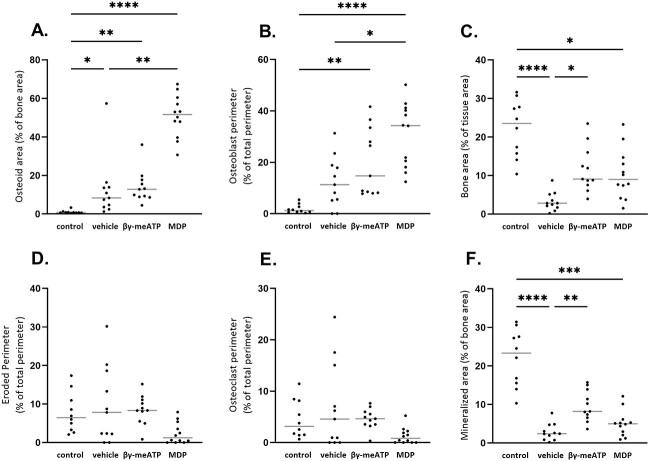
The effect of daily treatment with β,γ-methylene-ATP (β,γ-meATP) and medronic acid (MDP) treatment on static bone parameters in CKD rats. (A) Osteoid area, (B) osteoblast perimeter, (C) bone area, (D) eroded perimeter, (E) osteoclast perimeter, and (F) mineralized area. Data are presented as individual values (dots) and median (gray lines). ^*^p < .05, ^*^^*^p < .01, ^*^^*^^*^p < .001, and ^*^^*^^*^^*^p < .0001. Control (*n* = 10), vehicle (*n* = 11), β,γ-meATP (*n* = 11), and MDP (*n* = 12).

**Figure 8:**
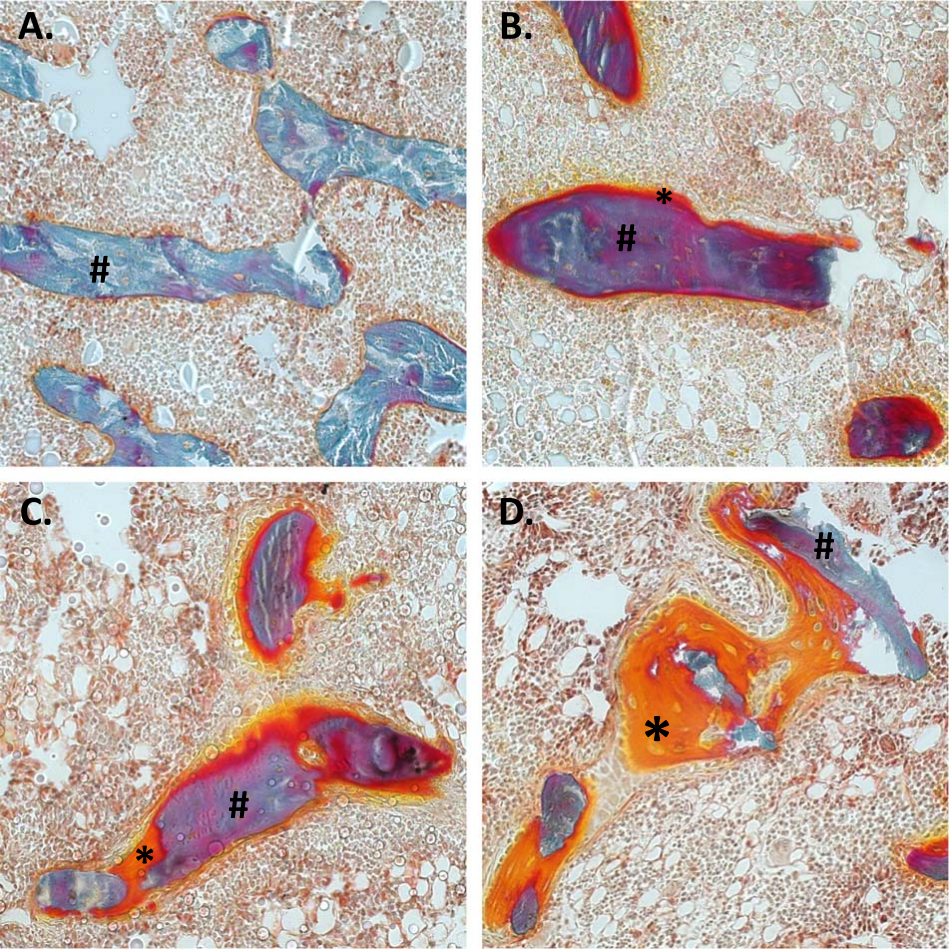
The effect of daily treatment with β,γ-methylene-ATP (β,γ-meATP) and medronic acid (MDP) on osteoid area. Representative Goldner-stained tibia sections of (A) control, (B) vehicle, (C) β,γ-meATP, and (D) MDP-treated CKD rats. Hashtag (#) indicates mineralized bone. Asterix (*) indicates osteoid or unmineralized bone. Images were taken at 20× magnification.

Since the processes of arterial media calcification and physiological bone mineralization have a high similarity and patients with CKD have an already compromised bone status, static bone parameters were investigated in all CKD groups. Figure 7 (A and B) shows that, in the presence of CKD, osteoid area and osteoblast perimeter increased versus that in control rats. However, these effects became significant when CKD rats were treated with β,γ-meATP (>10-fold increase in osteoid area vs control) and even more with MDP treatment (>50-fold increase in osteoid area vs control). This is also represented in Figure 8, which shows the microscopic images of Goldner-stained tibia sections. Furthermore, the bone and mineralized area was significantly decreased in all CKD rats versus control rats (Figure 7C). No significant differences were found between the groups for eroded perimeter and osteoclast perimeter, although a decreasing trend for these parameters was observed for CKD rats treated with MDP (Figure 7D and E).

## Discussion

Nucleotides are not only located intracellularly but cells have the capacity to release nucleotides into the extracellular space through controlled release or via cell death. The half-life of extracellular nucleotides is short, in the order of seconds, due to the close proximity of numerous ectonucleotide pyrophosphatase/phosphodiesterase (ie, NPPs) and therefore the paracrine radius is only a few hundred microns.^20^ To circumvent this short half-life and to selectively trigger the activation of specific P2 receptor subtypes, ATP analogs were developed including β,γ-meATP. The present study investigated the effect of β,γ-meATP and its metabolite MDP on arterial media calcification in a warfarin (non-CKD) and adenine (CKD) rat model. Consistent with the in vitro results of Patel et al*,*^16^ using a rat model of warfarin-induced arterial calcification, we for the first time show that a daily i.p. injection of β,γ-meATP adequately prevented the development of calcification in the aorta and peripheral vessels. This is evidenced by a significant reduction in the calcium content in these vessels and in the percentage of calcified area on Von Kossa–stained aortic sections.

High levels of serum calcium and phosphorus are important risk factors/triggers of the arterial calcification process. However, no differences in serum calcium and phosphorus levels were found between β,γ-meATP– and vehicle-treated rats exposed to warfarin, which might suggest that β,γ-meATP mediates its inhibitory effects by directly targeting the key players in the arterial calcification process (ie, VSMCs). These vascular cells undergo a phenotypic switch into osteo-/chondrogenic cells; however, β,γ-meATP did not significantly alter the mRNA expression of osteo-/chondrogenic marker genes *Alpl* (encoding TNAP) and *Sox9* in the aorta. Next to this, VSMCs regulate the local concentrations of the calcification inhibitor PP_i_ and stimulator P_i_ (PP_i_/P_i_ ratio) through the expression of ecto-nucleotide pyrophosphatase/phosphodiesterases (NPPs) and TNAP enzymes.^21^ NPP1 mediates the breakdown of ATP into AMP and PP_i_; however, it can also generate ADP and P_i_.^22^^,^^23^ Conversely, TNAP favors the production of P_i_ from PP_i_ and to a lesser extent from ATP.^22^^,^^23^ Furthermore, β,γ-meATP has been shown to inhibit NPP enzymes.^24^^,^^25^ In our warfarin study, no significant effect of β,γ-meATP on aortic mRNA expression of NPPs (*Enpp1* and *Enpp3*) could be detected. Still, a trend was observed in which the mRNA expression of *Sox9, Alpl, Enpp1*, and *Enpp3* was closer to control levels (wherein warfarin-induced calcification was inhibited). Since β,γ-meATP is a P2X1 receptor agonist, its gene expression was investigated in the aortic wall of warfarin-exposed rats. Similar to the in vitro data of Patel et al*,*^16^ no upregulation of the P2X1 receptor was observed for β,γ-meATP–treated rats. Also, in vitro data showed that P2X antagonists did not influence the VSMC mineralization process, suggesting a rather limited role for these receptors in arterial media calcification.^16^ An additional mechanism by which β,γ-meATP blocks arterial media calcification includes its breakdown by NPPs into a bisphosphonate-like compound, MDP.^24^^,^^25^ At high concentrations, bisphosphonates have anti-mineralizing effects by preventing the incorporation of P_i_ into the calcium-phosphate crystals and thus interfering with hydroxyapatite crystal formation and maturation.^26^ Hence, β,γ-meATP might also affect hydroxyapatite crystal deposition in the bone. Although in our warfarin study, only a modest effect of β,γ-meATP therapy on bone metabolism was observed, the osteoid area of β,γ-meATP–treated rats was significantly lower than that of vehicle-treated rats but equal to that of control rats fed the standard feed-pellet diet. In fact, β,γ-meATP treatment of warfarin-exposed rats returned the osteoid area values to control levels observed in healthy rats. Taken together, these promising findings were an impetus to further investigate the anti-arterial media calcification effect of β,γ-meATP and its metabolite MDP in a CKD setting.

Here, an adenine-induced CKD rat model was used, which is more physiological relevant (ie, driven by the complex CKD pathology instead of the absence of 1 single calcification inhibitor) and develops more severe arterial calcification compared with the warfarin rat model. Therefore, the dosage of β,γ-meATP was doubled in the CKD-related arterial media calcification study. Unfortunately, we observed that neither β,γ-meATP nor its metabolite MDP was able to significantly prevent the development of arterial media calcifications in CKD rats. A shortcoming of this study is the high variability of arterial media calcification development, which is inherent to animal models of CKD-related media calcification.^27^ This probably contributed to the lack of significant effect of both compounds on CKD-induced arterial media calcification. In fact, for the β,γ-meATP treatment group, individual aortic calcification scores were close to control values, with the exception of 1 animal. Moreover, treating CKD rats with β,γ-meATP or MDP prevented the upregulation of the osteo-/chondrogenic marker *Sox9*, indicating that both compounds affect osteogenic conversion of VSMCs. The lack of effect on *Alpl* expression is probably attributable to the fact that aortic TNAP overexpression precedes the calcium deposition. Hence, *Alpl* is an early marker for arterial calcification.^28^

We observed that the presence of CKD lowers aortic Enpp1 levels, probably making the vessel wall more prone to calcify (less local PPi production). Moreover, the calcium content in the thoracic aorta correlated positively and significantly with *Enpp1* and *Enpp3* aortic mRNA gene expression in vehicle-treated CKD rats and, as such, can be seen as (1) a survival mechanism, increasing local PPi levels to target the present arterial calcifications, or perhaps (2) an adverse process, by depleting local ATP levels leading to less purinergic signaling. Indeed, for the latter aspect, we have recently shown that mice with a purinergic receptor P2Y_2_ deletion are more susceptible to the development of arterial media calcification compared with wild-type mice.^29^ Furthermore, in the context of valve calcification, overexpression of the NPP1 enzyme was observed in calcifying valve interstitial cells.^30^^,^^31^ Moreover, silencing *Enpp1* expression or using an NPP1 inhibitor, quinazoline-4-piperidine sulfamide, prevented the mineralization and the expression of osteo-/chondrogenic marker genes in aortic valve interstitial cells.^30^^,^^32^ On the other hand, knockout mouse experiments showed that the absence of NPP1 activity also promotes severe arterial media calcification due to inadequate production of PP_i_.^33^ Furthermore, the exact mechanism by which NPP3 affects arterial calcification is unknown. One study by Villa-Bellosta et al^25^ argues that NPP3 participates in PP_i_ hydrolysis into P_i_. Altogether, the role of NPP enzymes in arterial media calcification is highly speculative and more research is needed to understand the role of the expression/activity profile of NPP enzymes over time in the process of arterial media calcification. Interestingly, however, in the current study, the control level of aortic expression of *Enpp1* and *Enpp3* seemed to be associated with low calcification scores.

The fact that both compounds, β,γ-meATP and its metabolite, did not significantly block CKD-related arterial calcifications suggests the need for higher dosages in case of severe calcification development. However, the CKD-related arterial media calcification study revealed an imminent risk of both therapies on physiological bone mineralization. In our CKD rat model, renal insufficiency favored a significant decrease in bone and mineralized area, whereas osteoid area was significantly augmented versus that in control animals. This histological bone profile corresponds to a mixed bone lesion, combining a high bone turnover with an osteomalacic component due to inappropriate hydroxyapatite crystal deposition by osteoblasts. Also, in the human situation, patients with CKD often experience this type of bone disease, which goes along with a high fracture risk.^34^ Moreover, MDP rather than β,γ-meATP worsened these CKD-related bone mineralization defects, with a significant elevation of the osteoid area and osteoblast perimeter, while mineralized area was significantly decreased. This is consistent with previous studies that have shown that ATP analogs and bisphosphonates inhibit osteoblast-mediated mineralization of the bone matrix.^11^^,^^26^

In conclusion, daily injections of the ATP analog β,y-meATP prevented the development of moderate, non–CKD-related arterial media calcifications, while its actions were inadequate to block severe, CKD-related arterial media calcifications. Moreover, β,y-meATP and its metabolite MDP induced deleterious effects on bone mineralization in rats with CKD, limiting the possibility to increase the dosage of both compounds—again, pointing out the difficult task of targeting solely ectopic calcifications without negatively affecting physiological bone mineralization. In the future, more attention needs to be paid to finding target-specific therapies against arterial media calcification (ie, the use of nanoparticles decorated with ligands that specifically target the vessel wall).

## Data Availability

The data underlying this article are available in the article. More detail about the datasets will be made available upon request.
